# Expression of AQP5 and AQP8 in human colorectal carcinoma and their clinical significance

**DOI:** 10.1186/1477-7819-10-242

**Published:** 2012-11-13

**Authors:** Wei Wang, Qing Li, Tao Yang, Guang Bai, Dongsheng Li, Qiang Li, Hongzhi Sun

**Affiliations:** 1 Department of General Surgery, The First Affiliated Hospital, Liaoning Medical University, No 2,5th Duan, People's Street, Guta District, Jinzhou, 121001, China; 2 Department of Renal Medicine, The Third Affiliated Hospital, Liaoning Medical University, Jinzhou, 121001, China

**Keywords:** AQP5, AQP8, Colorectal cancer, Immunohistochemistry

## Abstract

**Background:**

The aquaporins (AQPs) are a family of small membrane transport proteins whose overexpression has been implicated in tumorigenesis. However, the expression of AQP5 and AQP8 in colorectal cancer and the clinical significance remain unexplored. This study aimed to detect the expression of AQP5 and AQP8 in clinical samples of colorectal cancer and analyze the correlations of their expression with the clinicopathological features of colorectal cancer.

**Methods:**

Forty pairs of colorectal cancer tissue and paraneoplastic normal tissue were obtained at the time of surgery from patients with colorectal cancer. The expression of AQP5 and AQP8 was detected by immunohistochemical staining and reverse transcriptase polymerase chain reaction.

**Results:**

AQP5 was mainly expressed in colorectal carcinoma cells and barely expressed in paraneoplastic normal tissues. By contrast, AQP8 was mainly expressed in paraneoplastic normal tissues and barely expressed in colorectal carcinoma cells. AQP5 expression was not significantly associated with the sex or age of the patient with colorectal cancer (*P*>0.05), but was closely associated with the differentiation, tumor-nodes-metastasis stage and distant lymph node metastasis of colorectal carcinoma (*P*<0.05).

**Conclusions:**

AQP5 might be a novel prognostic biomarker for patients with colorectal cancer.

## Background

Colorectal carcinoma (CRC) is one of the most common malignancies in the world. Despite the advances in the diagnosis and treatment of CRC in the past several years, the overall prognosis for patients with CRC remains poor [1]. Therefore, many investigations have focused on the identification of novel prognostic and predictive biomarkers for CRC and a variety of biomarkers that could predict the survival of CRC have been reported recently [2-6].

It is well-known that tumor growth, development, invasion and metastasis depend on nutrient supply and metabolism and water molecules play an important role in the modulation of the tumor microenvironment and tumor metabolism. The aquaporins (AQPs) consist of a family of small membrane transport proteins. Up to now, 13 members of the AQPs have been identified in human, which can be subdivided into two groups based on the basis of their permeability: AQP1, 2, 4, 5 and 8 function primarily as water-selective transporters, while AQP3, 7, 9 and 10, termed aquaglyceroporins, transport water as well as glycerol and other small solutes [7]. The overexpression of several members of the AQPs family has been shown to be implicated in tumorigenesis. For example, AQP5 is overexpressed in breast cancer, cervical cancer and ovarian cancer [8-10]. However, the expression of AQP5 and AQP8 in CRC and their clinical significance remain unexplored. To evaluate the potential of AQP5 and AQP8 as novel prognostic markers of CRC, we employed RT-PCR and immunohistochemical methods to detect the expression of AQP5 and AQP8 in clinical samples of CRC and then analyzed the correlations of their expression with the clinicopathological features of CRC.

## Methods

### Patients and tumor samples

From December 2009 to January 2011, samples were collected from 40 cases of CRC from the Department of Surgery, the First Affiliated Hospital of Liaoning Medical University. The clinical samples were obtained from surgically removed, pathologically confirmed CRC. The patients included 25 men and 15 women and their ages ranged from 35 to 80 years old. None of the patients accepted preoperative radiotherapy or chemotherapy and none of them had hypertension or diabetes. The pathological classifications of CRC were based on tumor size, infiltration, differentiation and lymph node metastasis. The matched non-tumor adjacent tissue was obtained from a segment of the resected specimens that was the farthest from the tumor (>3 cm). Tissue samples were snap-frozen in liquid nitrogen and stored at −80°C until further analysis. The collection of clinical samples was approved by the Ethics Committee of Liaoning Medical University, and all patients gave their written informed consent.

### Immunohistochemical staining

Tumor tissues and normal tissues were fixed in 10% formaldehyde and were paraffin wax-embedded. Subsequently, the tissues were cut into 5-μm-thick serial sections, which were washed carefully with 0.01 M PBS three times. To block endogenous peroxidases, de-waxed paraffin sections were treated with 3% hydrogen peroxide for 20 min. The sections were blocked with 2% goat serum in 0.01 M PBS containing 0.3% Triton X-100 for 1 h at room temperature, then incubated at 4°C overnight with rabbit antibody against human AQP5 (1:250 dilution, Bioss Inc., Beijing, China) or AQP8 (1:200 dilution, Bioss Inc.). Afterwards, the sections were subjected to immunohistochemical staining using PV6000 kit (Bioss Inc.). Finally the sections were developed with diaminobenzidine and assessed by two independent investigators under light microscopy. The staining was considered to be positive when a strong coloration was evident in the membranes of the epithelial cells. Tissues were scored semi-quantitatively by evaluating the percentage of stained cells of 10 separate fields. For negative controls, the primary antibodies were replaced with PBS.

### RT-PCR

Total RNA was extracted from the tissues (100 g) using Trizol (Invitrogen, Carlsbad, CA, USA) following the manufacturer's protocol. cDNA was produced by reverse transcription using an RT kit (Takara, Shiga, Japan) following the manufacturer's protocol. PCR amplification of AQP5, AQP8 and β-actin was done with Taq Master Mix(Promega, Madison, WI, USA)with cDNA synthesized from the tissues. The primers used were as follows: AQP5 5^′^-TGACGAGGACTGGGAGGAGC-3^′^ (forward) and 5^′^-GGCGGCATTCAATGAACCA-3^′^ (reverse), amplicon 488 bp; AQP8 5^′^-TCATTGGAGATGGGAAGACC-3^′^ (forward) and 5^′^-TGAGAAGCAAGGAAGTGGC-3^′^ (reverse), amplicon 437 bp; β-actin 5^′^-GTTCGCCATGGATGACGATATC-3^′^ (forward) and 5^′^-GCCAGATCTTCTCCATGTCGTC-3^′^ (reverse), amplicon 128 bp. Amplification conditions were as follows: 5 min at 95°C (one cycle) and 40 s at 94°C; 40 s at the annealing temperature (58.5°C for AQP5, 56.8°C for AQP8 and 58.8°C for β-actin); and 1 min at 72°C (35 cycles) and 72°C for 5 min (one cycle). PCR products were analyzed by 2% gel electrophoresis following densitometric analysis of band intensity. The relative mRNA levels of AQP5 and AQP8 were determined with β-actin as the internal control.

### Statistical analysis

The data were expressed as mean (X) ± standard deviation (S). Statistical analysis was performed using SPSS13.0 software (SPSS, Inc., Chicago, IL, USA) and *P* <0.05 was considered significant.

## Results

### Immunohistochemistry

**Figure 1:**
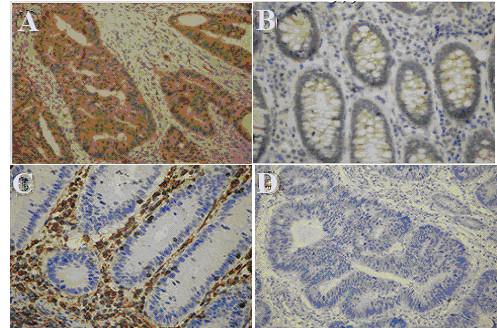
**Representative images of immunohistochemical staining to detect the expression of AQP5 and AQP8 in clinical colorectal samples.** (**A**) AQP5 immunoreactivity was detected in the cytoplasm and plasma membrane of colorectal carcinoma cells (dark brown). (**B**) AQP5 immunoreactivity was not detected in the adjacent normal colon epithelium. (**C**) AQP8 immunoreactivity was detected in the cytoplasm and plasma membrane of adjacent normal colon epithelium (dark brown). (**D**) AQP8 immunoreactivity was not detected in the colorectal carcinoma tissues. (Magnification: ×400).

Immunohistochemical staining detected AQP5 immunoreactivity in the cytoplasm and plasma membrane of colorectal carcinoma cells, but not in the adjacent normal colorectal tissues. By contrast, AQP8 immunoreactivity was detected in the cytoplasm and plasma membrane of adjacent normal colon epithelium, but was not detected in the colorectal carcinoma tissues (Figure 1).

### RT-PCR

**Figure 2:**
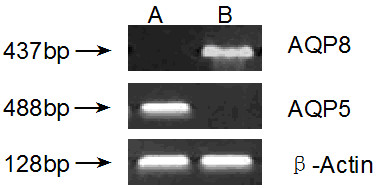
**Representative PCR analysis to detect the expression of AQP5 and AQP8 in clinical colorectal samples.** (**A**) Adjacent normal colon tissue. (**B**) Colorectal carcinoma tissue. AQP5 mRNA was not detected in adjacent normal colon tissue but was abundant in colorectal carcinoma tissue. By contrast, AQP8 mRNA was abundant in adjacent normal colon tissue but was not detected in colorectal carcinoma tissue. β-actin served as the internal control.

Next we performed RT-PCR analysis to detect the expression of AQP5 and AQP8 at mRNA level in clinical colorectal samples. The results showed that AQP5 mRNA was abundant in colorectal carcinoma tissue but undetectable in adjacent normal colon tissue. By contrast, AQP8 mRNA was abundant in adjacent normal colon tissue but undetectable in colorectal carcinoma tissue (Figure 2). These data are consistent with the results of immunohistochemical staining and confirm that the expression of AQP5 is upregulated but that of AQP8 is downregulated in colorectal carcinoma.

### The relationship of AQP5 expression with clinicopathological features of colorectal carcinoma

To investigate the clinical significance of AQP5 expression in colorectal carcinoma tissues, we analyzed the clinicopathological parameters of the patients. Statistical analysis showed that AQP5 expression was not significantly associated with the sex or age of the patients with CRC (*P*>0.05), but was closely associated with the differentiation, tumor-nodes-metastasis (TNM) metastasis stage and distant lymph node metastasis of colorectal carcinoma (*P*<0.05) (Table 1).

**Table 1:** The relationship between AQP5 expression in colorectal carcinoma and clinicopathological features

**Group**	**n**	**AQP5 expression**		***χ*2**	***P***
		**+**	**-**		
**Gender**					
Male	25	13	12	0.11	>0.5
Female	15	7	8		
**Age**					
≥50 years	28	14	14	0.23	>0.5
<50 years	12	7	5		
**Differentiation**					
High and medium	26	18	8	4.66	<0.5
Low	14	5	9		
**TNM stage**					
I + II	23	10	13	6.16	<0.5
III + IV	17	14	3		
**Lymphatic metastasis**					
Yes	21	7	14	4.91	<0.5
No	19	13	6		

## Discussion

Aquaporins play a crucial role in maintaining water homeostasis and glycerol metabolism and modulating a variety of physiological and pathological processes [7]. Significantly, several members of the AQPs family are involved in tumorigenesis, as suggested by their overexpression in clinical cancer tissues and cancer cell lines [8-10].

AQP5 has been shown to be overexpressed in several types of cancers and contribute to tumor growth, progression and metastasis. Consequently, AQP5 has been proposed as a putative oncogene [11,12]. Recently, Zhang *et al*. reported that AQP5 overexpression was significantly associated with lymph node involvement and a poorer prognosis in patients with cervical cancer, suggesting the value of AQP5 as a prognostic marker in cervical cancer [9]. Interestingly, Fischer *et al*. performed differential display to find that AQP8 mRNA was expressed only in normal colonic tissue and not, or to a much lesser extent, in the adenomas, carcinomas and cancer cell lines. *In situ* hybridization demonstrated that AQP8 was expressed in the cells facing the lumen in the normal colonic epithelium [13].

The clinical significance of AQP5 and AQP8 in colorectal carcinoma remains largely unexplored. We employed RT-PCR and immunohistochemical methods to detect the expression of AQP5 and AQP8 in clinical samples of colorectal carcinoma and the paraneoplastic normal colonic tissues. Our results showed that AQP5 was mainly expressed in colorectal carcinoma cells and barely expressed in paraneoplastic normal tissues. By contrast, AQP8 was mainly expressed in paraneoplastic normal tissues and barely expressed in colorectal carcinoma cells. The expression patterns of AQP5 and AQP8 in colonic tissues we detected are consistent with the results of previous studies [13,14]. Furthermore, we analyzed the correlations of AQP5 expression with the clinicopathological features of CRC and found that AQP5 expression was not significantly associated with the sex or age of patients with CRC (*P*>0.05), but was closely associated with the differentiation, TNM stage and distant lymph node metastasis of colorectal carcinoma (*P*<0.05). Taken together, our results demonstrate that AQP5 is upregulated and AQP8 is downregulated in CRC tissues.

It has been proposed that the expression of AQP8 is a marker of normal proliferating colonic epithelial cells and suggests these cells to be involved in fluid transport in the colon [13]. Alternatively, *in situ* hybridization demonstrated that the expression of AQP5 was induced in early-stage disease (early dysplasia) and then maintained through the late stages of colon cancer development [15]. Therefore, our observations that AQP8 expression was not detected in colorectal carcinoma while AQP5 expression was closely associated with the differentiation, TNM stage and distant lymph node metastasis of colorectal carcinoma suggest that the loss of AQP8 expression and gain of AQP5 expression is involved in the process of colorectal carcinogenesis.

Notably, it was reported recently that epigallocatechin gallate, a potential anticancer drug, inhibited the proliferation and induced the apoptosis of ovarian cancer SKOV3 cells through the downregulation of AQP5 expression [16]. This study confirmed the overexpression of AQP5 in CRC and suggested that AQP5 may be a potential therapeutic target. Further studies are important to characterize the potential oncogenic role of AQP5 and antitumor role of AQP8 in CRC development. Larger samples should be examined to validate the value of AQP5 as a novel prognostic biomarker for patients with CRC.

## Conclusion

AQP5 may be a novel prognostic biomarker for patients with colorectal carcinoma.

## Competing interests

The authors declare that they have no competing interests.

## Authors' contributions

WW and TY carried out the main experiments. QingL, GB and DL collected the samples. QiangL performed the statistical analysis studies. HS conceived the study and drafted the manuscript. All authors read and approved the final manuscript.
